# Proton Pump Inhibitor-Associated Hypomagnesemia: A Case Report Highlighting a Rare Adverse Effect

**DOI:** 10.7759/cureus.115974

**Published:** 2026-09-08

**Authors:** Hugo Sabillon, Anna Fuentes, Isabella Novoa, Jorge Novoa

**Affiliations:** 1 University School of Health Sciences, Universidad Nacional Autónoma de Honduras, San Pedro Sula, HND; 2 Faculty of Medicine, Universidad Católica de Honduras, San Pedro Sula, HND; 3 Internal Medicine, Cemesa Hospital, San Pedro Sula, HND

**Keywords:** chronic therapy with ppis, drug induced hypomagnesemia, electrolyte disorders, hypocalcemia, hypomagnesemia, ppi-associated hypomagnesemia, proton pump inhibitors (ppis)

## Abstract

Proton pump inhibitors (PPIs) are widely prescribed for the management of gastric acid-related diseases, such as gastroesophageal reflux disease (GERD), and are generally well tolerated, with few or mild adverse effects. We present a case of an elderly female patient who developed hypomagnesemia, a rare but serious complication associated with chronic PPI therapy. The patient had been receiving esomeprazole 40 mg daily for approximately 12 months. She developed secondary electrolyte disorders, with hypocalcemia being the most evident. Her clinical manifestations initially included muscle cramps and tremors, which progressed to a seizure and eventually to altered mental status with a positive Trousseau sign. Despite institutional constraints, PPI-associated hypomagnesemia was considered, and the patient improved following electrolyte replacement and discontinuation of esomeprazole. Because fractional excretion of magnesium could not be calculated, the mechanism of magnesium loss could not be definitively established. This report highlights the importance of considering PPI-associated hypomagnesemia, an infrequent but potentially serious adverse effect, in patients receiving prolonged PPI therapy who develop compatible clinical manifestations.

## Introduction

Proton pump inhibitors (PPIs) are drugs that irreversibly inhibit the hydrogen-potassium ATPase (H⁺/K⁺-ATPase) pump in the parietal cells of the stomach, thereby reducing gastric acid secretion [1]. PPIs are widely prescribed for the management of gastric acid-related diseases such as peptic ulcer disease, erosive esophagitis, and gastroesophageal reflux disease (GERD) [2]. Although PPIs are generally intended for short-term use, their prolonged and, in some cases, lifelong use has become increasingly common. Chronic use of PPIs may lead to several adverse effects, including enteric infections, renal damage, bone fractures, and various nutritional deficiencies. Among these, hypomagnesemia is an uncommon but potentially serious complication of long-term PPI use [2].

Hypomagnesemia is primarily related to impaired magnesium absorption in the gastrointestinal (GI) tract. However, the exact mechanism remains unknown. Evidence suggests that an increase in the GI luminal pH induced by PPIs results in reduced Mg²⁺ solubility, thereby impairing its absorption in the small intestine [3]. In addition, this increase in pH reduces the activity of melastatin-type transient receptor potential (TRPM) channels 6 and 7, resulting in reduced transcellular Mg²⁺ transport in the colon. Another mechanism involves changes in the composition and function of the gut microbiome [3].

Hypomagnesemia can be associated with secondary electrolyte disturbances (hypokalemia, hypocalcemia, hyponatremia), cardiac arrhythmias, seizures, and other neuromuscular disorders [4]. In this report, we describe a case of hypomagnesemia associated with prolonged esomeprazole therapy. The report underscores the diagnostic challenge posed by severe hypomagnesemia, which initially manifested with neurological symptoms and significant hypocalcemia before the underlying magnesium deficiency was identified.

## Case presentation

A 69-year-old female patient presented to the emergency department following one episode of a generalized tonic-clonic seizure. On interrogation, she reported a history of type 2 diabetes mellitus, hypertension, chronic GERD, hiatal hernia, and chronic normocytic normochromic anemia. Her chronic medications included linagliptin, insulin glargine, telmisartan, atorvastatin, esomeprazole 40 mg once daily, and oral ferrous sulfate and folic acid. She had been taking esomeprazole for approximately 12 months. Her anemia had been present for approximately two years and had partially improved with iron and folic acid supplementation; however, its underlying etiology had not been definitively documented. She also complained of short-term memory loss, tremors, and cramping, especially in her hands and lower limbs, for the two months preceding her emergency department visit. Her vital signs were normal, and physical examination revealed no abnormalities.

Initially, a complete blood count was performed, which demonstrated normocytic normochromic anemia (Table 1). Additional laboratory testing revealed a reduced total serum calcium level (Ca²⁺ total: 6.3 mg/dL), with a serum potassium level of 3.5 mEq/L and a glucose level of 80 mg/dL. Brain MRI showed mild-to-moderate cortical and subcortical atrophic changes and chronic ischemic changes, without evidence of acute or subacute ischemia, intracranial hemorrhage, or intracranial mass lesion. Serum magnesium was not measured during the initial presentation, as the diagnostic evaluation focused on the identified hypocalcemia. Consequently, the patient received intravenous calcium gluconate and, after clinical improvement, was discharged. The underlying cause of the hypocalcemia, including possible hypomagnesemia, was not identified at that time.

**Table 1:** Complete blood count on initial admission MCV: mean corpuscular volume; MCHC: mean corpuscular hemoglobin concentration; WBC: white blood cells

Parameter	Patient value	Reference value
Hemoglobin (g/dL)	8.4	11.2-15.7
MCV (fL)	87.7	79.4-94.8
MCHC (g/dL)	32.2	32.2-35.5
Hematocrit (%)	26.2	34.1-44.9
WBC (10^3^/μL)	13.7	3.98-10.04

Three days later, the patient returned to the emergency room with altered mental status; she was somnolent, poorly responsive, and confused, and physical examination revealed a positive Trousseau sign (carpal spasm). Further laboratory analysis demonstrated low serum magnesium (Mg: 0.5 mg/dL), potassium (K⁺: 3 mEq/L), and total serum calcium (Ca²⁺ total: 7.6 mg/dL) (Table 2), along with phosphorus (P⁺: 4.6 mg/dL), parathyroid hormone (PTH: 19.1 pg/mL), and 25-hydroxyvitamin D (31.5 ng/mL).

**Table 2:** Serial serum and urine electrolyte measurements during hospitalization and follow-up “Not measured” indicates that the laboratory measurement was not obtained at that clinical time point UMG: random urine magnesium concentration

Parameter	First hospitalization: Day 1	Second hospitalization: Day 1	Second hospitalization: Day 2	After discharge: Day 2	After discharge: Day 4	Reference values
Ca^2+^total (mg/dL)	6.3	7.6	8.5	9.2	10.1	8-10
Mg(mg/dL)	Not measured	0.5	1.1	1.8	1.8	1.6-2.5
K^+ ^(mEq/L)	3.5	3	4.6	4.6	5	3.5-5.5
UMG (mg/dL)	Not measured	2.6	3.65	Not measured	Not measured	0.4-15

To assess renal magnesium loss, fractional excretion of magnesium (FEMg) was requested; however, it could not be performed due to institutional constraints. Only a random urine magnesium concentration (UMG) was obtained (UMG: 2.6 mg/dL). Since a random urine magnesium concentration alone cannot definitively distinguish between renal and gastrointestinal magnesium loss, the mechanism of magnesium loss could not be established. Considering the prolonged use of esomeprazole and the clinical findings, PPI-associated hypomagnesemia was considered the most likely cause. The patient received intravenous electrolyte replacement therapy, resulting in clinical improvement. Additionally, esomeprazole was discontinued and replaced with famotidine. By hospital day two, repeat laboratory tests showed that her electrolyte levels were beginning to normalize (Table 2). After complete recovery, she was discharged with oral therapy (calcium carbonate and magnesium chloride).

Follow-up laboratory tests performed during the four days following discharge showed sustained improvement (Table 2). At approximately six months after discharge, the treating physician reported that the patient remained clinically stable and that follow-up laboratory parameters remained within normal limits, allowing discontinuation of calcium supplementation. The exact laboratory values from this later follow-up were not available to the authors. Although definitive causality could not be established, the prolonged exposure to esomeprazole, together with the clinical and biochemical course, supported PPI-associated hypomagnesemia as the most plausible diagnosis. Causality was further assessed using the Naranjo Adverse Drug Reaction Probability Scale [5]. The patient had a total score of 7, corresponding to a probable adverse drug reaction (ADR) (Table 3).

**Table 3:** Naranjo Adverse Drug Reaction Probability Scale applied to the present case ^*^Rechallenge with esomeprazole was not performed because re-exposure after severe symptomatic hypomagnesemia was considered clinically and ethically inappropriate. According to the detailed instructions for the Naranjo scale, a “Yes” response may be assigned when the causal association is well known and rechallenge could not be performed for clinical or ethical reasons [5] A total Naranjo score of 7 indicates a probable adverse drug reaction. According to the Naranjo scale, scores ≥ 9 are classified as definite, 5-8 as probable, 1-4 as possible, and ≤ 0 as doubtful [5]

Question		Yes	No	Do not know	Score
1	Are there previous conclusive reports on this reaction?	+1	0	0	1
2	Did the adverse event appear after the suspected drug was administered?	+2	-1	0	2
3	Did the adverse event improve when the drug was discontinued, or a specific antagonist was administered?	+1	0	0	1
4	Did the adverse event reappear when the drug was readministered?	+2	-1	0	2^*^
5	Are there alternative causes that could on their own have caused the reaction?	-1	+2	0	0
6	Did the reaction reappear when a placebo was given?	-1	+1	0	0
7	Was the drug detected in blood or other fluids in concentrations known to be toxic?	+1	0	0	0
8	Was the reaction more severe when the dose was increased or less severe when the dose was decreased?	+1	0	0	0
9	Did the patient have a similar reaction to the same or similar drugs in any previous exposure?	+1	0	0	0
10	Was the adverse event confirmed by any objective evidence?	+1		0	1
Total score:					7

## Discussion

An ADR is defined by the World Health Organization as a noxious and unintended response to a medicine occurring at doses normally used in humans [6]. PPIs exert their effect by irreversibly inhibiting the H+/K+-ATPase pump in the gastric parietal cells, which are responsible for acid secretion. Inhibition of this enzyme leads to suppression of gastric acid secretion and an increase in gastric pH [1-2]. These drugs are widely used and considered the cornerstone of GERD treatment. Although generally well tolerated, they can occasionally be associated with minor side effects such as headache and GI upset. Serious side effects are less frequently observed and are more commonly associated with chronic PPI therapy. Nevertheless, a growing number of reported side effects have led to increasing concerns [7].

The major side effects associated with prolonged PPI therapy include cardiovascular events, kidney disease, Clostridium difficile infections, dementia, bone fractures, pneumonia, iron deficiency, vitamin B12 deficiency, and electrolyte disorders. Some of these effects are presumed to be a consequence of the suppression of gastric acid secretion [7-8]. Hypomagnesemia (serum magnesium < 0.7 mmol/L, approximately < 1.7 mg/dL) associated with prolonged use of PPIs is primarily related to impaired GI Mg²⁺ absorption. However, the exact mechanism remains unclear. Most evidence suggests that PPIs induce changes in GI luminal pH and the microbiome, decreasing Mg²⁺ solubility and thus its absorption [3,9]. Mg²⁺ absorption occurs in the GI tract via two main pathways: paracellular and transcellular. The paracellular pathway predominates in the small intestine, whereas in the colon, the main mechanism is transcellular transport, facilitated by TRPM6 and TRPM7 channels [3].

Inhibition of acid secretion by PPIs raises luminal pH, reducing Mg²⁺ solubility and thus its absorption in the GI tract. Moreover, increased pH may also reduce paracellular permeability and diminish the activity of TRPM6 and TRPM7 channels. Regarding the GI microbiome, it plays an important role in maintaining a low luminal colonic pH through fermentation. PPIs alter its composition, thereby impairing this function [3]. Patients who develop hypomagnesemia typically present with nonspecific symptoms, including muscle weakness, muscle cramps, or lethargy [9].

Hypomagnesemia results in a wide range of clinical manifestations, including cardiovascular effects due to the influence of Mg²⁺ on myocardial excitability. Among the most common electrocardiogram changes, moderate hypomagnesemia may manifest as peaked T waves and widening of the QRS complex, whereas severe hypomagnesemia usually presents with progressive widening of the QRS complex, prolongation of the PR interval, and diminution of the T wave. It has also been associated with an increased risk of atrial fibrillation and ventricular arrhythmias [10].

Hypomagnesemia is often accompanied by several electrolyte imbalances, such as hypokalemia resulting from increased renal potassium loss. Low Mg²⁺ also reduces the activity of the Na+Cl- cotransporter, which decreases sodium (Na+) reabsorption. In addition, it may interfere with PTH release, leading to secondary hypocalcemia [9]. Reaching an accurate diagnosis can be challenging due to the difficulty in suspecting Mg²⁺ abnormalities without specific clinical symptoms, which is why Mg²⁺ levels are usually measured in serum or plasma.

Given circadian variations in Mg²⁺ excretion, calculating urinary magnesium excretion together with serum or plasma magnesium levels provides a reliable approach for calculating fractional excretion and supporting an accurate diagnosis [11]. Institutional and economic impediments prevented some of these tests from being performed on the patient, thereby limiting timely diagnosis and treatment. In this case, FEMg could not be calculated, and the available random urine magnesium concentration was not sufficient to definitively distinguish renal from GI magnesium loss. Therefore, the mechanism of magnesium loss could not be established.

Furthermore, electrolyte replacement and discontinuation of esomeprazole occurred simultaneously, limiting definitive causal attribution. Although alternative etiologies could not be completely excluded and definitive causality cannot be established from a single case, the prolonged exposure to esomeprazole, severe hypomagnesemia with associated electrolyte abnormalities, and subsequent clinical and biochemical improvement support PPI-associated hypomagnesemia as the most plausible diagnosis. Routine magnesium monitoring in all patients receiving long-term PPI therapy is not universally recommended [7]; however, regulatory guidance suggests considering serum magnesium measurement before and periodically during prolonged PPI therapy [12]. Treatment relies on electrolyte replacement therapy and discontinuation of PPI therapy [13], which was successfully implemented in the present case with satisfactory results.

## Conclusions

PPIs are broadly used, and adverse effects are generally uncommon and, when they occur, usually mild. Nevertheless, serious adverse events may occasionally arise. This case report highlights the occurrence of a rare but potentially serious electrolyte disorder, PPI-associated hypomagnesemia, in a patient receiving prolonged PPI therapy. Although definitive causality cannot be established from a single case, the clinical and biochemical course supports an association between prolonged PPI use and hypomagnesemia. It also underscores that serum magnesium monitoring may be considered during prolonged PPI therapy, given the nonspecific clinical presentation and potentially severe consequences of hypomagnesemia.
